# A Ln(iii)-3-hydroxypyridine pH responsive probe optimized by DFT†

**DOI:** 10.1039/c9ra11058e

**Published:** 2020-03-03

**Authors:** Michael A. Caldwell, Christopher R. Brue, Tyler J. Whittemore, Thomas J. Meade

**Affiliations:** Departments of Chemistry, Molecular Biosciences, Neurobiology, and Radiology, Northwestern University Evanston IL 60208 USA tmeade@northwestern.edu +1 847 491 2481

## Abstract

Differences in tissue pH can be diagnostic of cancer and other conditions that shift cell metabolism. Paramagnetic probes are promising tools for pH mapping *in vivo* using magnetic resonance spectroscopy (MRS) as they provide uniquely shifted MR signals that change with pH. Here, we demonstrate a 3-hydroxy-6-methylpyridyl coordinating group as a new pH-responsive reporter group for Ln(iii) MRS probes. The pH response of the complex was observed by UV-Vis, fluorescence, and NMR spectroscopies, and modeled using DFT. These results provide insight into the observed pH-dependent NMR spectrum of the complex. The protonation state of the hydroxypyridine changes the coordinating ability of the ligand, affecting the dipolar field of the lanthanide and the chemical shift of nearby reporter nuclei. The favorable pH response and coordination properties of the 3-hydroxypyridyl group indicate its potential for further development as a dual responsive-reporter group. Incorporation into optimized scaffolds for MRS detection may enable sensitive pH-mapping *in vivo*.

## Abstract 2

The favorable pH response and coordination properties of the 3-hydroxypyridyl group indicate its potential for further development as a dual responsive-reporter group for Ln(iii) MRS probes.

## Graphical abstract

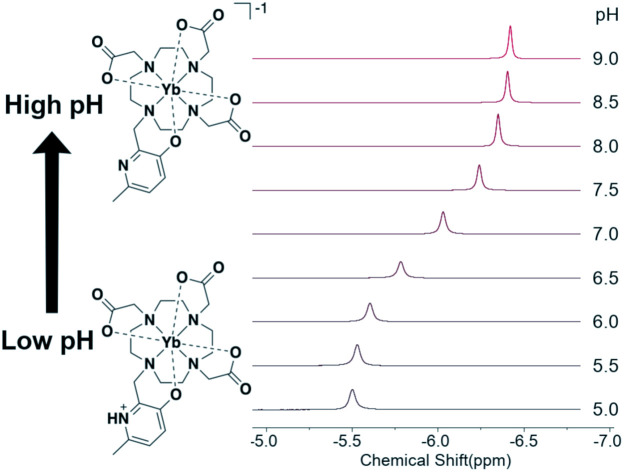

## Introduction

Dysregulation of extracellular pH is a hallmark of solid tumors and inflammation. In a solid tumor, poor vascularization and metabolic changes lead to anoxia and the accumulation of anaerobic metabolites, decreasing the local pH.^1,2^ Inflammatory signaling and decreased pH in the tumor microenvironment have been shown to limit the efficacy of the immune response and to accelerate metastatic and invasive potential.^2,3^ Directly monitoring and mapping pH *in vivo* provides an important tool to detect, monitor, and treat these conditions, and significant advances have been made in recent decades developing optical and magnetic resonance (MR) probes to measure extracellular pH.^4–10^ MR spectroscopy (MRS) techniques are increasingly utilized for these applications because they can non-invasively examine pH-dependent signals of an array of metabolites and probes with high spatial and spectral resolution.^5,6,11^

Paramagnetic metals, particularly lanthanides, impute valued properties for MR probes. Interactions with the unpaired spins of the metal reduce magnetic relaxation times and impact the resonant frequencies of nearby nuclei producing unique signals.^9,12–15^ These properties have been applied to monitor solution pH through relaxometry,^12,16,17^ paramagnetic chemical exchange saturation transfer (paraCEST),^13,18–20^ and chemical shift imaging.^15,21–23^ Generally, these probes respond to pH by varying structure, exchange rates, and/or protonation state resulting in changing signal intensity or frequency.^13–15,17^ For Ln(iii) chemical shift probes, an NMR-active reporter group is fixed at an appropriate distance from the metal to produce bright signal with a unique chemical shift.^15,22–26^ Incorporation of multiple reporter signals has been shown to increase the linear response range and decrease the impact of factors such as temperature through ratiometric or relative chemical shift measurements of multiple reporter signals.^21,22,27^ A responsive group is used to perturb the ligand field of the metal and/or the position of the reporter group to generate a change in the chemical shift of the reporter group in response to stimuli.^15,28–30^ A wide variety of protic groups have been applied as responsive groups for Ln(iii) pH-sensing, notably substituted phenols^10,23,31–33^ and phosphates.^22,25,27^ As imaging probes become increasingly multifunctional, moieties that can serve as both reporters and responsive groups are increasingly needed to enable incorporation of additional functionalities for ratiometric imaging or targeting.

In this investigation we evaluate a hydroxymethylpyridine (HMP) moiety as a dual reporter-responsive group for pH measurement. Evaluation of Ln(iii)-DO3A-HMP enables focused study of the behavior of HMP in a well-characterized ligand system (DO3A). The pH response of the complex between pH 6 and pH 8 can be observed by the changing absorption spectra of the ligand, decreasing Eu(iii)-centered luminescence intensity, and pH-dependent chemical shift of the pyridyl methyl group. Concomitant structural and electronic changes have been modeled with DFT. These results show meaningful perturbation of reporter NMR chemical shifts due to the changing electron donating ability of HMP upon deprotonation. HMP is a promising modular reporter-responsive group for Ln(iii) MRS probes and promises to enable new multifunctional probes for sensitive pH monitoring *in vivo*.

## Experimental

### General materials and methods

Unless otherwise noted, materials and solvents were obtained from Alfa Aesar (Haverhill, MA) or Sigma-Aldrich (St. Louis, MO) and used without further purification. 1,4,7,10-Tetraazacyclododecane (cyclen) was obtained from Strem Chemicals (Newburyport, MA) and used without further purification. Unless specified, all reactions were performed under dry nitrogen atmosphere. Thin layer-chromatography spots were visualized using cerium ammonium molybdate (CAM), potassium permanganate, ninhydrin, and potassium tetrachloroplatinate(ii) stains prepared according to lab SOPs. Reagent grade dichloromethane, dimethylformamide, acetonitrile, tetrahydrofuran, and triethylamine were dried using a Glass Contour solvent system.

NMR spectra were acquired on a 400 MHz Bruker Avance III HD Nanobay system equipped with SampleXpress autosampler unless otherwise noted. For measurements at non-ambient temperatures, samples were allowed to equilibrate in the magnet for ≥3 minutes. ESI-MS spectra were acquired on a Bruker AmaZonSL system equipped with a quadropole ion trap and Agilent 1100 quad pump system. All fluorescence measurements were made using a Hitachi F4500 fluorometer (Schaumburg, IL) with excitation wavelength 310 nm, scan rate 240 nm s^−1^, excitation slit width 10 nm, and emission slit width 5 nm. Each spectrum is the average of 3 scans. All UV-visible spectra were recorded using an Agilent Cary 60 UV-Vis system in dual beam mode with scan rate 600 nm min^−1^ and are the average of three scans.

Preparative HPLC was performed on an Agilent 1260 Infinity semi-prep system with a PrepStar 218 solvent delivery module, a 1260DAD VL diode array detector, and a 440LC fraction collector. A Waters 19 × 250 mm Atlantis T3 prep column was used for separations. Analytical HPLC was performed on Agilent 1260 Infinity II LC system equipped with a 1260 Infinity II Quaternary Pump, an inline diode array UV-Vis detector and Agilent 6120 Quadrupole LCMS System with a Waters 4.6 × 250 mm 5 μm Atlantis T4 column. HPLC-grade acetonitrile (Solvent B) and deionized water (Solvent A) were used for the mobile phase. Deionized water was obtained from a Millipore Q-guard system equipped with a quantum Ex cartridge.

### Potentiometric titrations

pH adjustment was conducted using a VWR SB21 pH meter and concentrated (0.5 M or 0.01 M) NaOH and HCl solutions to keep total change in volume <5%. For optical measurements, a solution of 0.125 mg mL^−1^ Eu(iii)-DO3A-HMP in 0.1 M NaCl was titrated with nitrogen bubbling to exclude ambient CO_2_. At each point, aliquots were removed for UV-Vis and fluorescence measurements and returned.

For NMR measurements, an approximately 8 mg mL^−1^ solution of Yb(iii)-DO3A-HMP or Y(iii)-DO3A-HMP in 0.1 M NaCl with 10% D_2_O as a lock solvent, and 1 mM DSS as an internal standard was titrated at 35 °C. At each point, an aliquot was removed from the titration and inserted into the magnet where it was allowed to equilibrate at 35 °C for 3 minutes. Spectra were acquired using a default presat sequence for water suppression. Acq. time and d1 were set to 0.5 s. 128 scans were acquired at each with ∼1 : 45 s in total acquisition time. These measurements were performed in triplicate.

To determine the p*K*_a_ of Ln(iii)-DO3A-HMP, a solution of 0.5 mg mL^−1^ Yb(iii)-DO3A-HMP in deionized water was prepared. 0.01 M NaOH and 0.1 M HCl solutions were standardized using potassium hydrogen phthalate and sodium carbonate, respectively. This measurement was performed in triplicate.

### Computational methods

All electronic calculations were performed using hybrid DFT with the B3LYP exchange correlation functional in the ORCA 4.0.1.2 package. Full geometry optimizations were performed first *in vacuo* on both the protonated and deprotonated complexes using TURBOMOLE def2-TZVPP triple-zeta basis set and the effective core potential (ECP) of Dolg *et al.*^34,35^ on all Yb(iii) atoms. Gas phase optimized structures were then further optimized using a conductor-like polarizable continuum model (CPCM) under the same conditions. The def2-TZVP triple-zeta basis set was used on all other C, H, N, and O atoms. RIJCOSX approximation was used with the def2/J auxiliary basis set.^36^ No symmetry constraints were used. SCF convergence criteria was 10–8 a.u. (“TightSCF”). Optimized geometries were then used in time-dependent DFT (TD-DFT) calculations of excited state transitions. The same basis sets and approximations were used in the TD-DFT calculations as were used in the geometry optimizations. The CPCM model of water was used as the solvent for these calculations to improve compatibility with the experimental UV-Vis data and 100 transition roots were calculated. Molecular orbital compositions and analyses were completed using the AOMix program.^37^

### Synthesis

#### Synthesis of 1,4,7-tris-*tert*-butoxycarbonylmethyl-1,4,7,10-tetraazacyclododecane (^*t*^Bu-DO3A)

The HBr salt of 1,4,7-tris-*tert*-butoxycarbonylmethyl-1,4,7,10-tetraazacyclododecane was synthesized according to the literature and is shown in ESI Scheme 1.†^38^ The free base was generated quantitatively by stirring the HBr salt with 1 equivalent of KOH in EtOH for one hour. Insoluble KBr was removed by filtration and the eluent was dried to yield 1 quantitatively.

#### Synthesis of 1,4,7-tris-*tert*-butoxycarbonylmethyl-10-(3-hydroxy-6-methylpyridylmethyl)-1,4,7,10-tetraazacyclododecane (^*t*^Bu-DO3A-HMP)

1 (500 mg, 0.87 mmol) was dissolved in toluene and 0.37% (w/w) formaldehyde in H_2_O (0.17 mL, 1.74 mmol) was added. The solution was stirred at 40–50 °C for 10 min before 3-hydroxy-6-methylpyridine was added (118 mg, 1.74 mmol). The reaction was stirred at 40–50 °C overnight and the solvent was removed. The crude product was purified by silica column in 5–10% MeOH/DCM (61% yield). ESI-MS *m*/*z* = 636.46 [M + H]^+^

#### Synthesis of 1,4,7-carboxymethyl-10-(3-hydroxy-6-methylpyridylmethyl)-1,4,7,10-tetraazacyclododecane (DO3A-HMP)

2 was dissolved in 30% TFA/DCM and stirred overnight. The solvent was removed, and the product was used without further purification. ESI-MS *m*/*z* = 499.00 [M + H]^+^

#### General procedure for metalation with LnCl_3_ (Ln(iii)-DO3A-HMP)

**Scheme 1:**
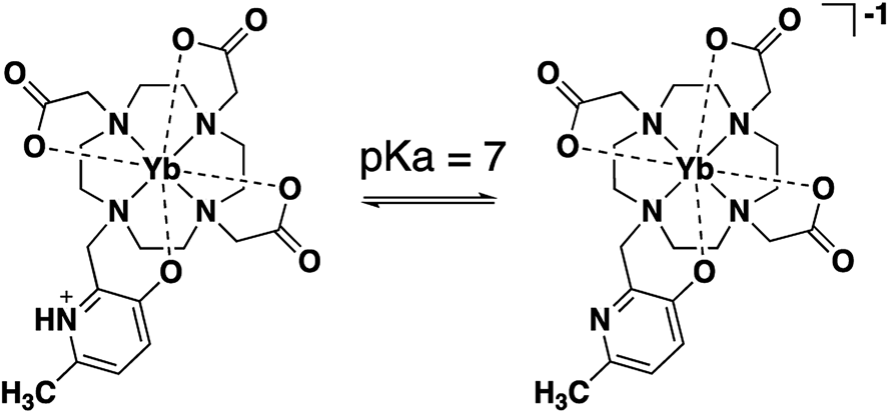
Deprotonation of HMP in Ln(iii)-DO3A-HMP between pH 6 and pH 8 increases the electron density of the coordinating oxygen, perturbing the ligand field, and leading to an enhanced change in the NMR shift of the pyridyl methyl group over a diamagnetic analog due to the shifting pseuodocontact field of the lanthanide.

Crude deprotected ligand was dissolved in deionized H_2_O and the pH was adjusted to 7. LnCl_3_ (2 equivalents) was added and the mixture was stirred overnight. The pH was raised to 10 to precipitate any remaining Ln(iii) as Ln(OH)_3_ and the solution was centrifuged. The pH of the supernatant was adjusted to 7 and the solution was filtered for HPLC purification (Scheme 1).

#### 1,4,7-Carboxymethyl-10-(3-hydroxy-6-methylpyridylmethyl)-1,4,7,10-tetraazacyclododecane yttrium(iii) (Y(iii)-DO3A-HMP)

HRMS (ESI) *m*/*z* = 554.1278 (calcd = 554.1274).

#### 1,4,7-Carboxymethyl-10-(3-hydroxy-6-methylpyridylmethyl)-1,4,7,10-tetraazacyclododecane europium(iii) (Eu(iii)-DO3A-HMP)

HRMS (ESI) *m*/*z* = 618.1427 (calcd = 618.1427).

#### 1,4,7-Carboxymethyl-10-(3-hydroxy-6-methylpyridylmethyl)-1,4,7,10-tetraazacyclododecane ytterbium(iii) (Yb(iii)-DO3A-HMP)

HRMS (ESI) *m*/*z* = 639.1612 (calcd = 639.1604).

## Results and discussion

### Synthesis of Ln(iii)-DO3A-HMP

Ln(iii)-DO3A-HMP was synthesized in three steps with high yield. Literature conditions^39^ for the addition of 3-hydroxy-6-methylpyridine to tetraazamacrocycles were modified to accommodate 1,4,7-tris-*tert*-butoxycarbonylmethyl-1,4,7,10-tetraazacyclododecane (^*t*^BuDO3A) and gave ^*t*^Bu-DO3A-HMP in high yield. ^*t*^Bu-DO3A-HMP was deprotected in acid and metallated with Ln(iii) (Y(iii), Eu(iii), and Yb(iii)) prior to facile HPLC purification.

The coordination of Ln(iii) by DO3A-HMP was evaluated by variable temperature NMR (VTNMR) analysis. The NMR spectrum of Yb(iii)-DO3A-HMP shows one major peak for the pyridyl methyl group near −6 ppm along with minor peaks, most notably at −8.5 ppm and at −73.13 ppm, indicating the presence of multiple conformations of Yb-DO3A-HMP in solution. The peak at −6 ppm constitutes ∼60% of the signal, indicating an excess of this conformer (ESI Fig. 2†). Similar patterns have been observed in the literature for 8-coordinate derivatives of DO3A.^19,40–43^ The chemical shift and linewidth of the major resonance at −6 ppm and minor resonance at −8.5 ppm show temperature dependence (ESI Fig. 6†). The linewidths of both decrease between 1 °C and 50 °C and increase significantly between 50 °C and 90 °C, indicating the presence of two exchange processes detectable by NMR. The observed broadening above 50 °C is consistent with reported isomerization processes for DO3A-based chelates, but occurs at a somewhat higher temperature than most reported complexes, potentially indicating enhanced rigidity of this system.^12,43,44^ The broadening at lower temperatures may be due to prototropic exchange at the hydroxypyridine nitrogen, but the broadening does not lead to a significant decrease in intensity at 35 °C compared to 50 °C (ESI Fig. 6†).

### The electronics of HMP change significantly between pH 6 and pH 8

**Fig. 1:**
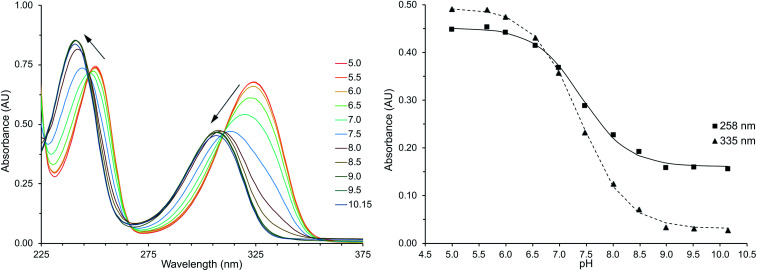
Left: the UV-Vis absorption spectrum of Eu(iii)-DO3A-HMP (100 μM, 0.1 M NaCl) hypsochromically shifts with increasing pH, consistent with deprotonation and attributed to greater electron density on the coordinating oxygen. Right: least squares fitting of absorption maxima (258 and 335 nm) *vs.* pH yields a p*K*_a_ of 7.4.

Deprotonation of HMP was observed by the hypsochromic shift in the pyridine based bands at 314 nm and 246 nm (Fig. 1), similar to previous studies.^45,46^ The magnitude of these observed shifts are consistent with TD-DFT calculations for the protonated and deprotonated species of Ln(iii)-DO3A-HMP (ESI Table 1†). Fitting the change in absorbance at wavelengths with large changes in absorptivity at low and high pH gives an apparent p*K*_a_ for this change of 7.4 in 0.1 M NaCl (Fig. 1, inset), consistent with the p*K*_a_ measured by potentiometric titration (ESI Fig. 1†). Strong electronic coupling between the hydroxyl group and the pyridyl nitrogen of HMP and large changes in basicity upon protonation or deprotonation have been reported,^10,45–47^ which we hypothesized would lead to variable coordination strength with pH.

### Eu(iii)-centered luminescence reflects changes in HMP coordination strength.

**Fig. 2:**
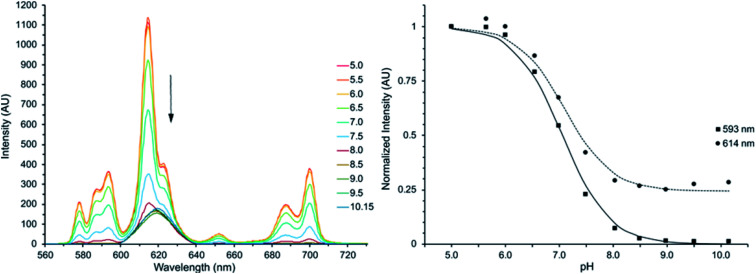
Left: the luminescence intensity of Eu(iii)-DO3A-HMP decreases with increasing pH in 0.1 M NaCl when excited at the UV-Vis isosbestic point of 310 nm. The approximate p*K*_a_ of 7, in agreement with UV-Vis and potentiometric measurements of the deprotonation of the HMP ring, indicates the Eu(iii) center is perturbed by changing coordination of the ligand anionic form of the ligand. Right: normalized intensities at 593 nm and 614 nm *vs.* pH. Lines represent least-square fits: p*K*_a_593__ = 7.06 p*K*_a_614__ = 7.10.

The intensity of all transitions in the emission spectrum of Eu(iii)-DO3A-HMP decreases in a pH-dependent manner until almost entirely quenched when irradiated at the transition isosbestic point (*λ*_ex_ = 310 nm) (Fig. 2). The putative mechanism for this quenching is the formation of a ligand–metal charge transfer (LMCT) state upon deprotonation to the phenoxide species. This mechanism is possible due to the relatively low reduction potential of Eu(iii) and has been observed for a number of coordinating phenoxides in the literature.^16,31–33^ LMCT-band energies of Eu(iii) are strongly affected by ligand electronegativity^30,32,48–51^ and the formation of the LMCT state is evidence for a significant change in the electron donating ability of the oxygen of the hydroxypyridyl ring upon deprotonation of the pyridyl nitrogen, consistent with direct deprotonation of a coordinating phenol.

### Computational analysis supports increased coordination strength upon deprotonation of HMP

The effects of ligand deprotonation were further evaluated using DFT and support tight Ln(iii) coordination by the deprotonated species. Orbital overlap between the HMP ligand and the Yb(iii) ion increases upon deprotonation. This is best seen in the overlap between the fragment orbitals of Yb(iii) and methyl reporter atoms, where the sum of the absolute values increases from 0.479 to 0.519 upon deprotonation. The distance between the coordinating phenolate oxygen and the Yb(iii) is larger in the protonated structure (2.2385 Å protonated *vs.* 2.2047 Å deprotonated) and the Mayer bond order, a measure of bond covalency, increases from 0.445 to 0.527 upon HMP protonation. This suggests that there is increased electronic coupling between the HMP ligand and the Yb(iii) ion in the deprotonated state.

### NMR monitoring of pH with Yb(iii)-DO3A-HMP

**Fig. 3:**
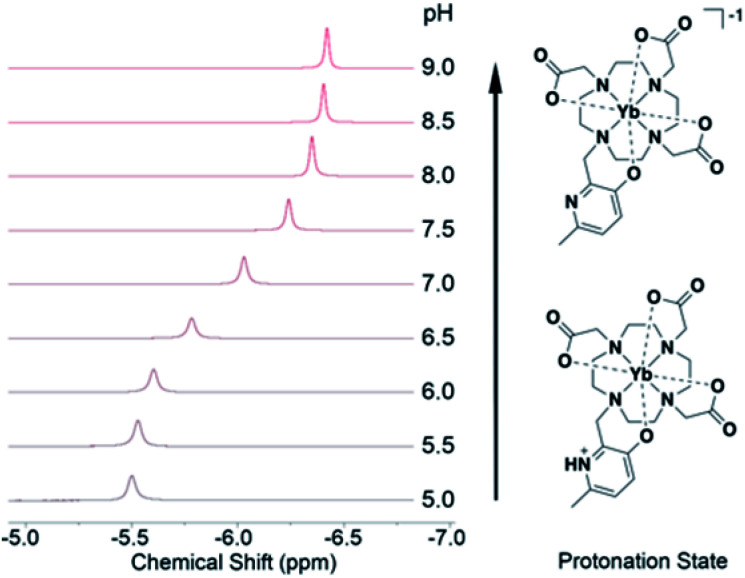
The chemical shift of the resonance of Yb-DO3A-HMP varies in concert with UV-Vis and luminescent changes across the range of pH 6 to pH 8 due to induced changes in the Yb(iii) dipolar field by changing HMP coordination upon deprotonation.

Tighter coordination by HMP results in changing NMR shifts, enabling tracking of pH. While the diamagnetic Y(iii)-DO3A-HMP also shows a pH dependent NMR signal, it is located near 2.5 ppm, a typically crowded region in ^1^H NMR and shifts upfield less than 0.2 ppm over the relevant pH range (ESI Fig. 4†). The resonance near −6 ppm of Yb(iii)-DO3A-HMP shows more than four-fold larger shift across this range (Fig. 3). This response is linear between pH 6.5 and 7.5 (*R*^2^ = 0.995), encompassing the majority of pHs observed in biological samples, and somewhat linear over the full range of 6–8 (*R*^2^ = 0.986) with a response of −0.4 ppm/pH. The linewidth of the major resonance decreases with increasing pH, particularly above the measured p*K*_a_, supporting the hypothesis that the observed decreases in linewidth up to 50 °C are due to prototropic exchange at the pyridyl nitrogen, which is not observed at high pH. However, the observed exchange broadening at 35 °C does not significantly degrade detection (ESI Fig. 6†).

## Conclusions

The hydroxymethylpyridyl responsive group evaluated here demonstrates efficient transmission of solution pH information to the Ln(iii) center and a linear change in the chemical shift of the paramagnetically-shifted methyl resonance over a biologically-relevant pH range. This group has been shown to bind lanthanides tightly and is easily added to tetraazamacrocycles using inexpensive reagents.^39^ Incorporating both reporter and responsive functionalities in a single coordinating group will enable inclusion of additional functions into the chelate for improved probe performance. Most notably, inclusion of an optimized reporter arm can provide a second, brighter reporter signal and enable measurement of relative peak intensities or shifts for improved probe performance.^15,21,22,27,29^ In addition, groups for targeting or conjugation could be incorporated to improve accumulation and half-life in tissues of interest. Further evaluation of this group for the design of modular, multifunctional imaging probes *in vivo* pH measurement is on-going.

## Author contributions

The manuscript was written by contributions of all authors.

## Funding sources

No competing financial interests have been declared.

## Abbreviations

MRS: Magnetic resonance spectroscopy
PARACEST: Paramagnetic chemical exchange saturation transfer
DO3A: 1,4,7-Triacetate-1,4,7,12-tetraazacyclododecane
HMP: Hydroxymethylpyridine
PRE: Paramagnetic relaxation enhancement
PCS: Psuedocontact shift

## Conflicts of interest

There are no conflicts to declare.

## Supplementary Material

RA-010-C9RA11058E-s001
