# Supplementary material for: A Ln(iii)-3-hydroxypyridine pH responsive probe optimized by DFT
Source: RSC Adv. 2020 Mar 3;10(15):8994–9. doi: 10.1039/c9ra11058e (PMC7144623; doi:10.1039/c9ra11058e)
Supplement: RA-010-C9RA11058E-s001 [file RA-010-C9RA11058E-s001.pdf]

Electronic Supplementary Material (ESI) for RSC Advances.
This journal is © The Royal Society of Chemistry 2020

Supplementary Information

A Ln(III)-3-hydroxypyridine pH responsive probe optimized by DFT

Michael A. Caldwell, Christopher R. Brue, Tyler J. Whittemore, and Thomas J. Meade*

Departments of Chemistry, Molecular Biosciences, Neurobiology and Radiology, Northwestern University, Evanston, IL, 60208.

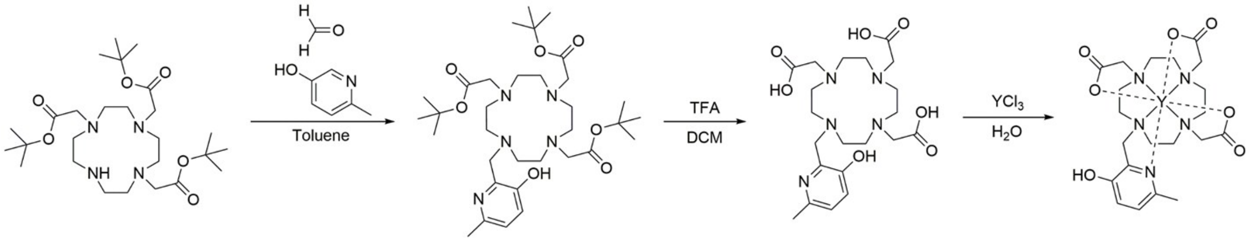

Supplementary Scheme 1. Synthesis of Y(III)-DO3A-HMP

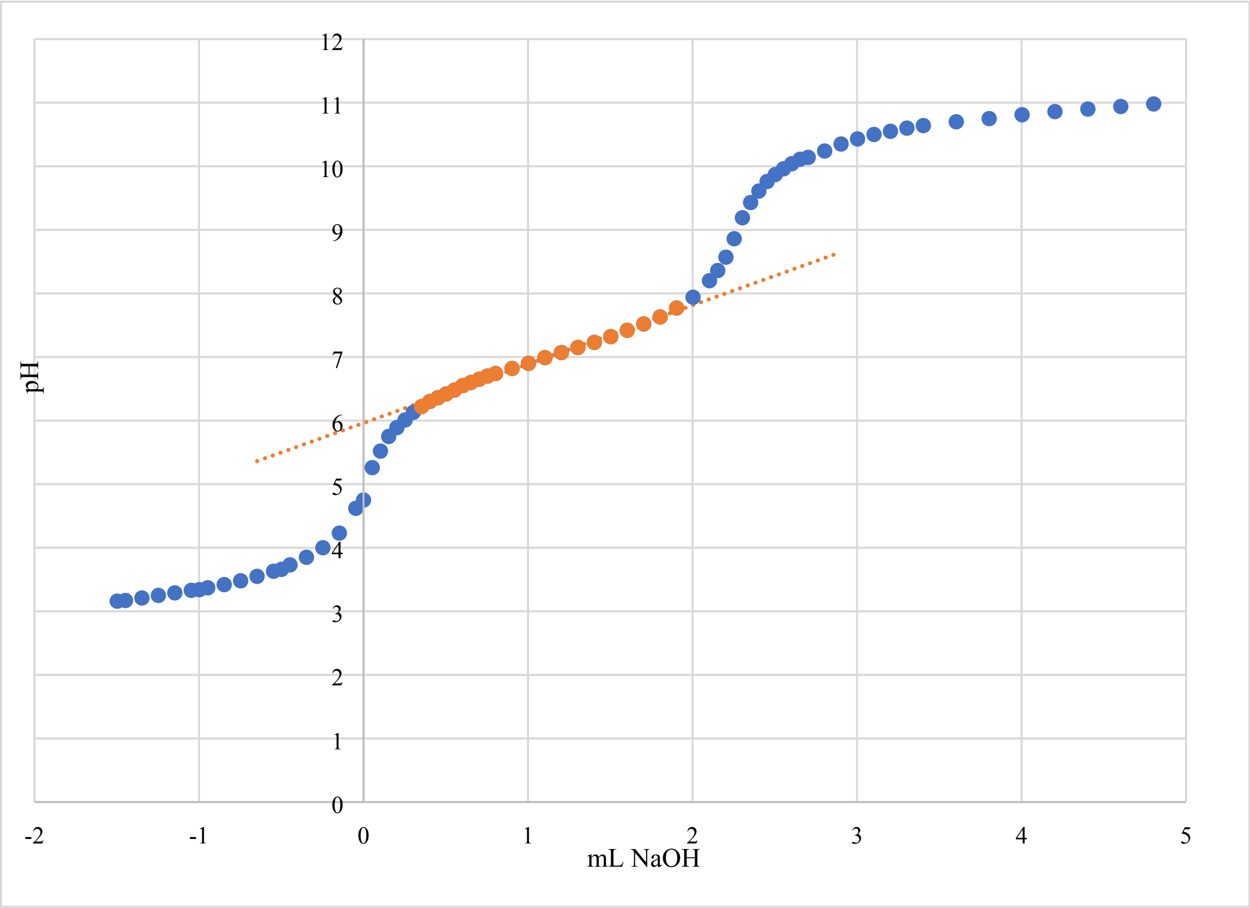

Supplementary Figure 1. Titration of Yb(III)-DO3A-HMP identified the pK_a of HMP in the complex to be 7 with a linear pH response between pH 6 and pH 8.

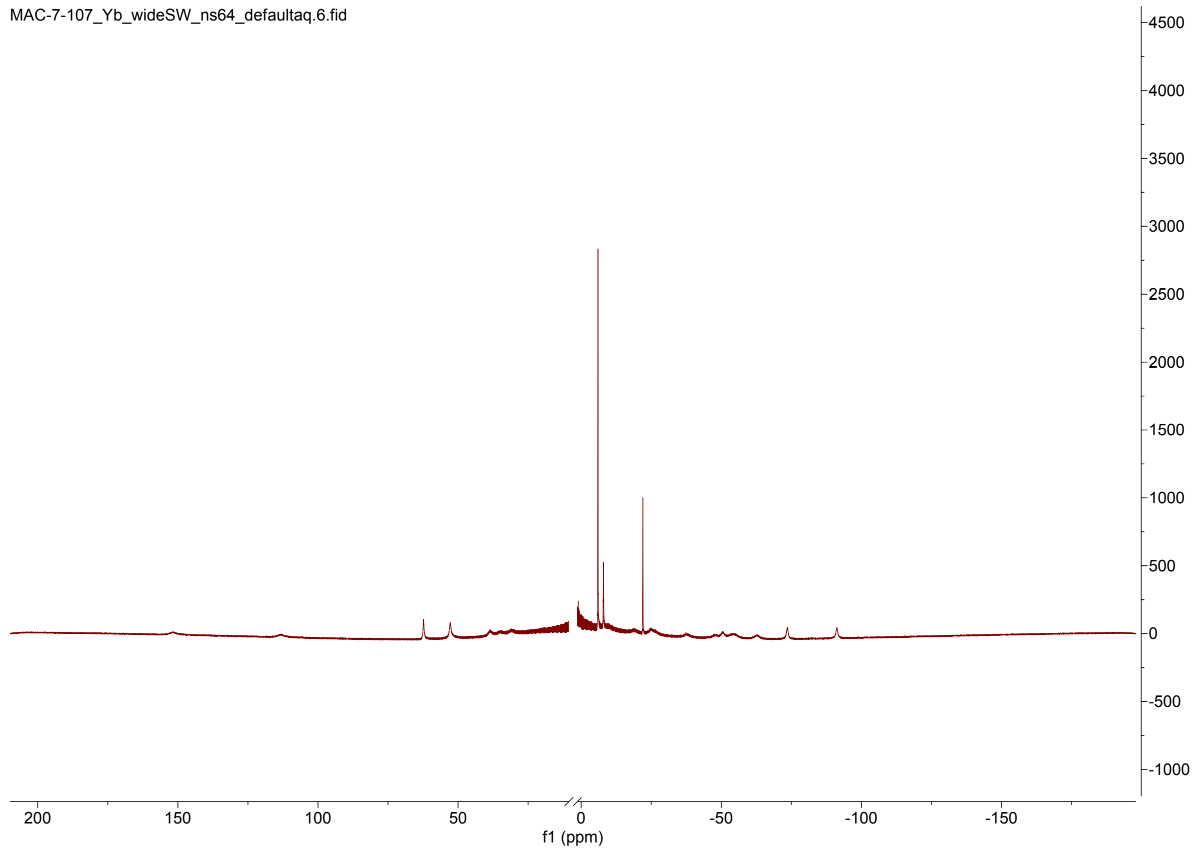

Supplementary Figure 2. Wide sweep width NMR of Yb(III)-DO3A-HMP shows the peak at -6ppm dominates the spectrum. The presence of small peaks above 100ppm and 150ppm are indicative of both SAP and TSAP isomers in solution.

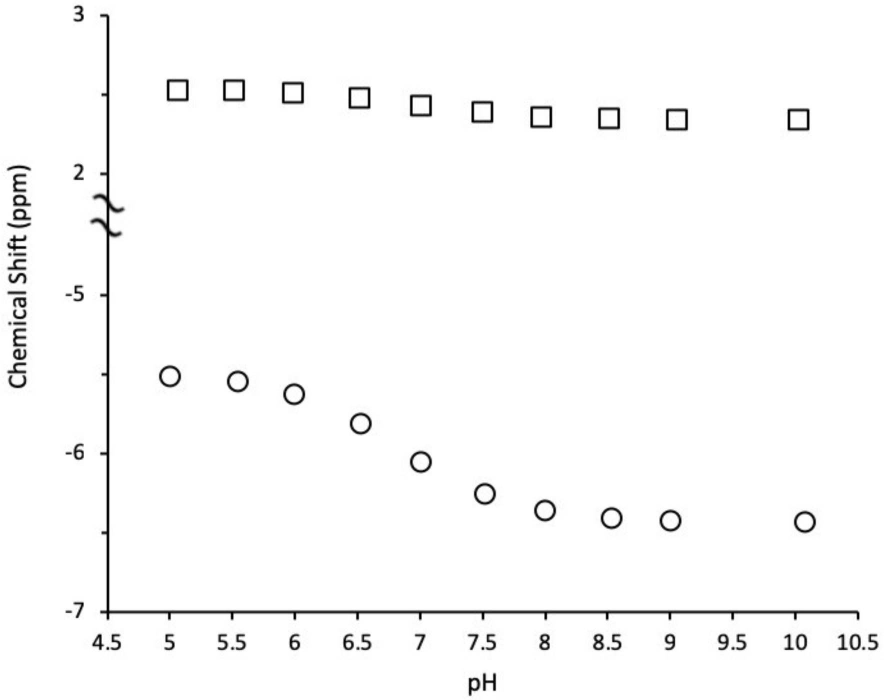

Supplementary Figure 3. The change in 1HNMR chemical shift of diamagnetic Y(III)-DO3A-HMP (top) versus Yb(III)-DO3A-HMP (bottom). While both show pH-dependence, the change in ligand electronics alone is not sufficient to explain the much larger change in the paramagnetic complex, supporting the role of the changing dipolar (psuedocontact) field of Yb(III) in the pH response of Yb(III)-DO3A-HMP.

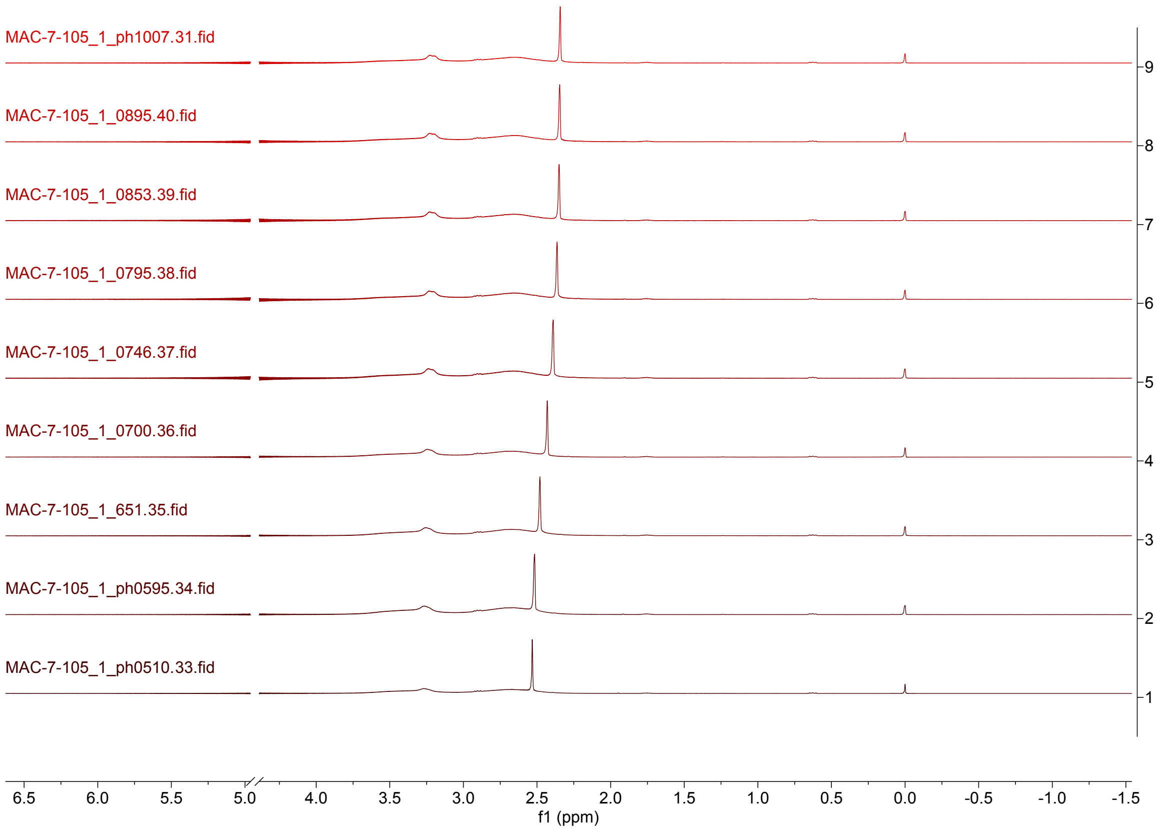

Supplementary Figure 4. Stacked NMR spectra of Y(III)-DO3A-HMP at varying pH show a very small change in chemical shift with pH.

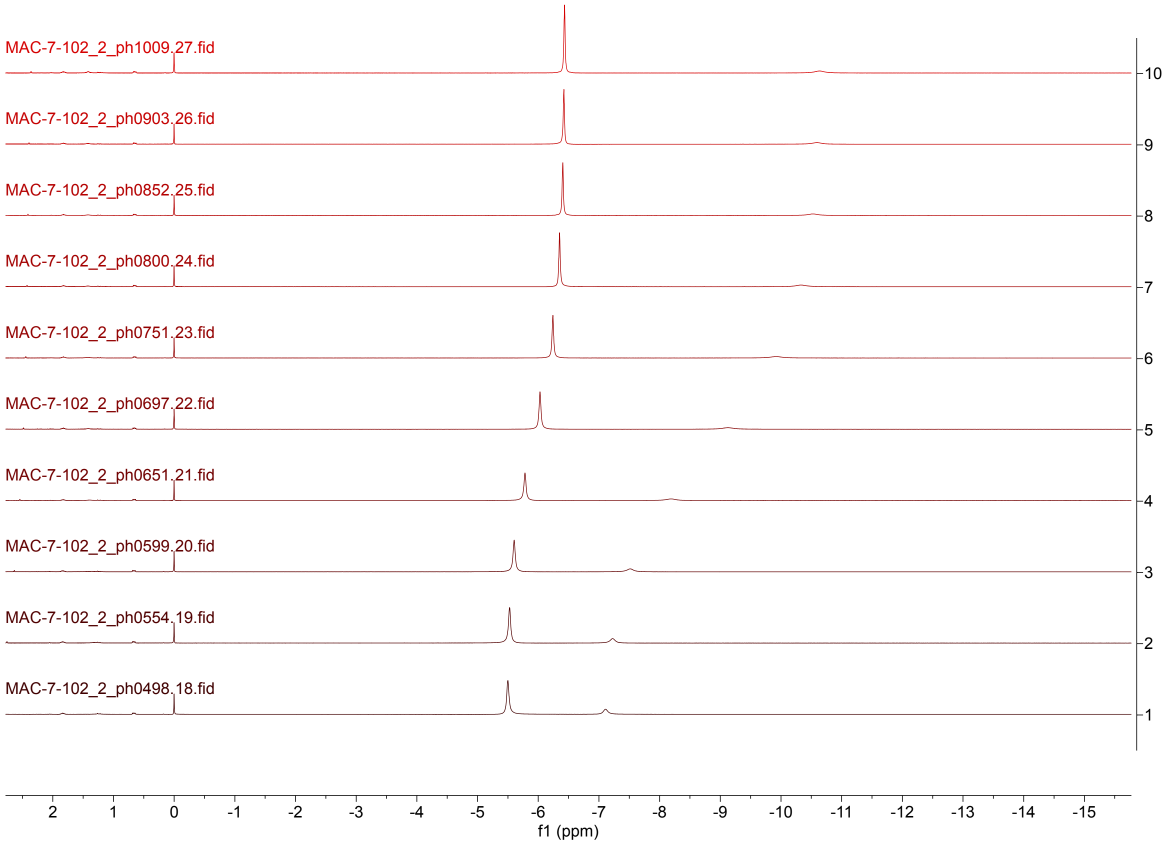

Supplementary Figure 5. Stacked NMR spectra of Yb(III)-DO3A-HMP at varying pH show a ~1ppm change in chemical shift and decreasing linewidth with pH.

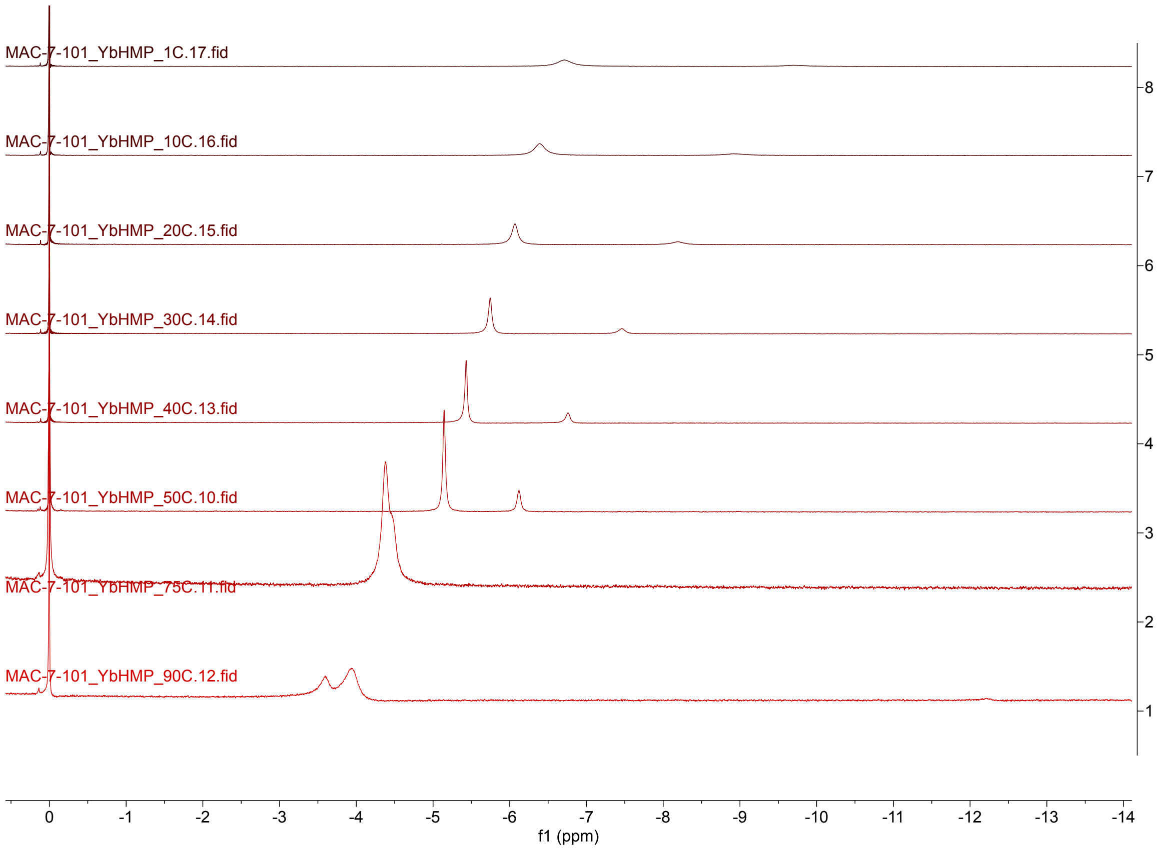

Supplementary Figure 6. VTNMR of Yb(III)-DO3A-HMP shows temperature-dependent changes in chemical shift and linewidth. The change in chemical shift is roughly linear, while two trends can be observed for the linewidth. First, the linewidth decreases between 1°C and 50°C. This is likely due to prototropic exchange from the protonated nitrogen of HMP, as similar decreases in linewidth are observed with increasing pH. Above 50°C, the linewidth begins to increase, likely due to increased conformational exchange of the macrocyclic ring and coordinating arms.

UV-VIS	T1 / eV	T2 / eV	TD-DFT	T1 / eV	T2 / eV
Deprotonated	5.10	4.01	Deprotonated	5.36	4.43
Protonated	4.92	3.78	Protonated	5.08	4.07
ΔE	0.18	0.23	Blueshift	0.28	0.36

Supplementary Table 1. The pyridine π→π* transitions that dominate the electronic absorption spectra show similar shift in the calculated and empirical spectra, where the major lowest energy transitions (T1, T2) show similar hysochromic shifts (ΔE) upon protonation (Table 1).

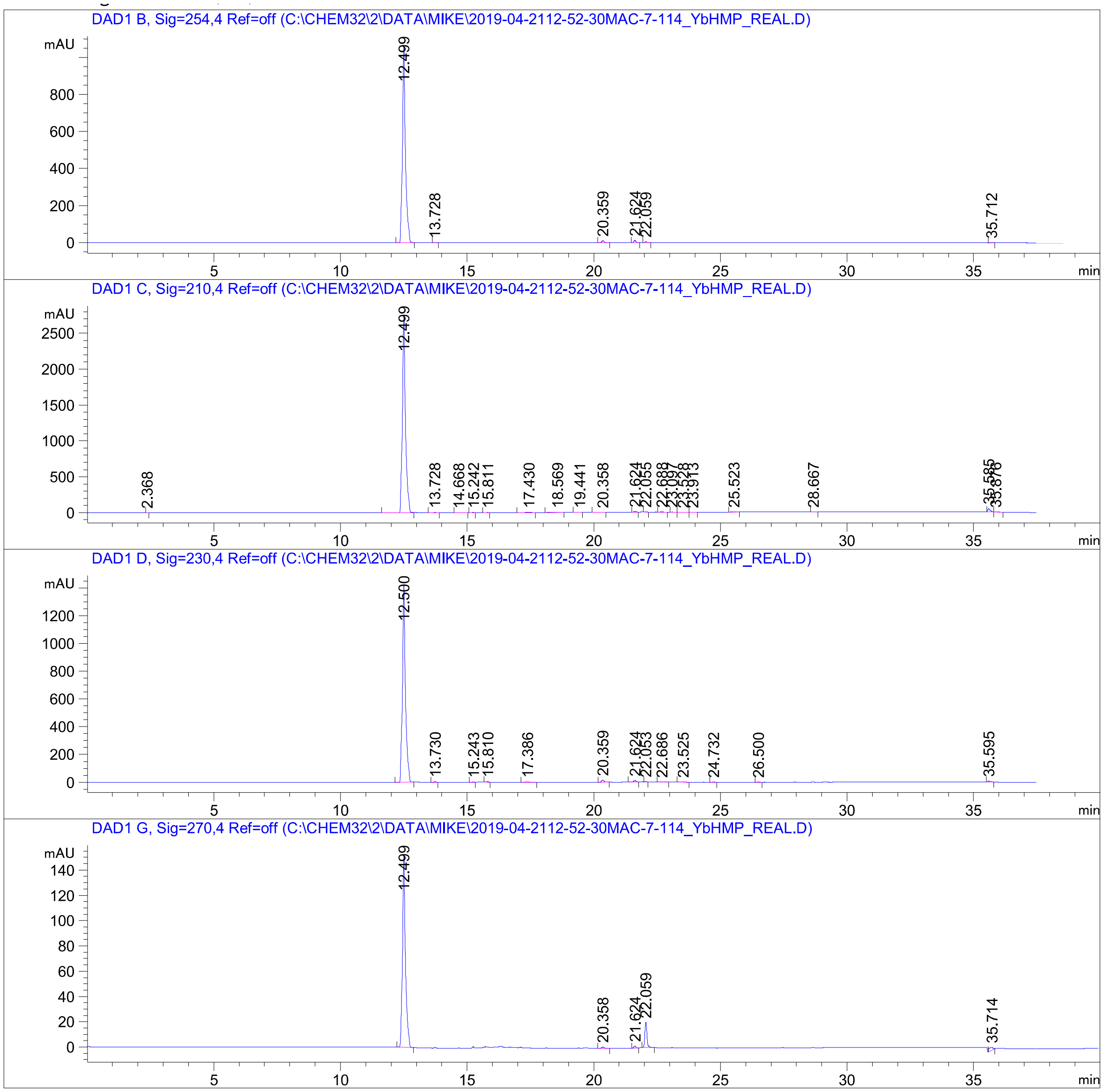

Supplementary Figure 7. Analytical HPLC trace of Yb(III)-HMP-DO3A at 254nm, 210nm, 230nm, and 270nm.

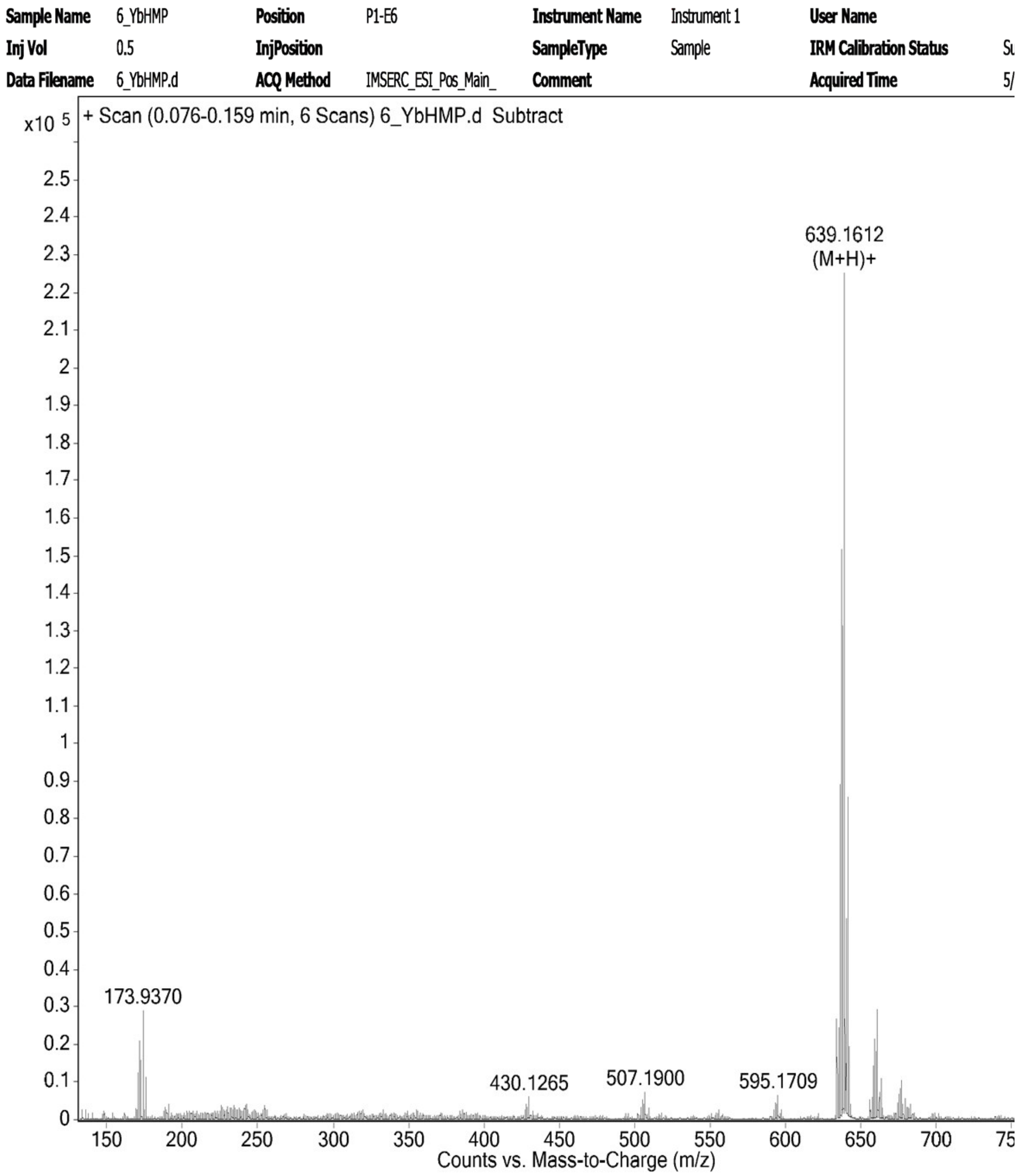

Supplementary Figure 8. HRMS of Yb(III)-HMP-DO3A.

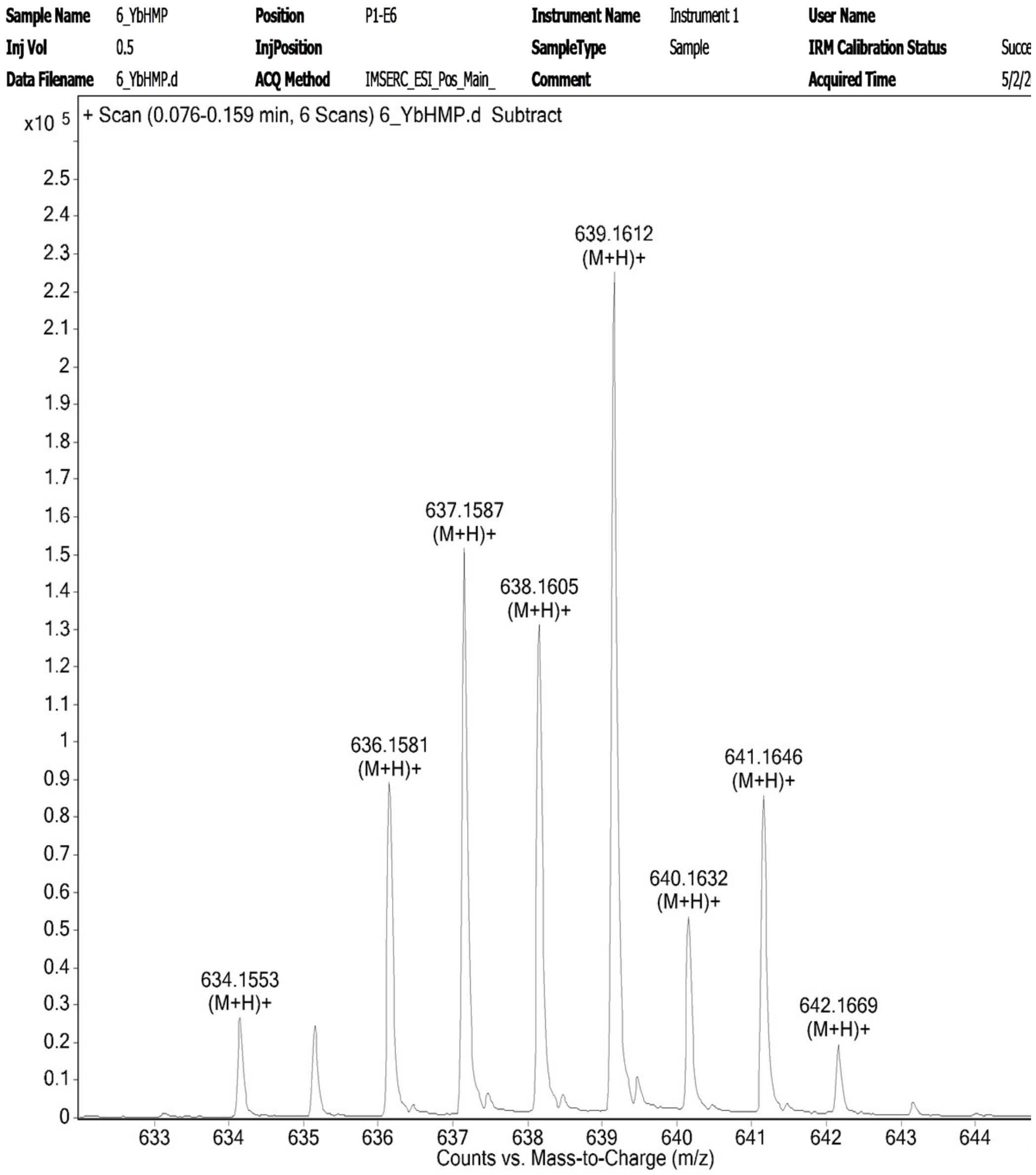

Supplementary Figure 9. HRMS of Yb(III)-HMP-DO3A showing the isotopic pattern of Yb.

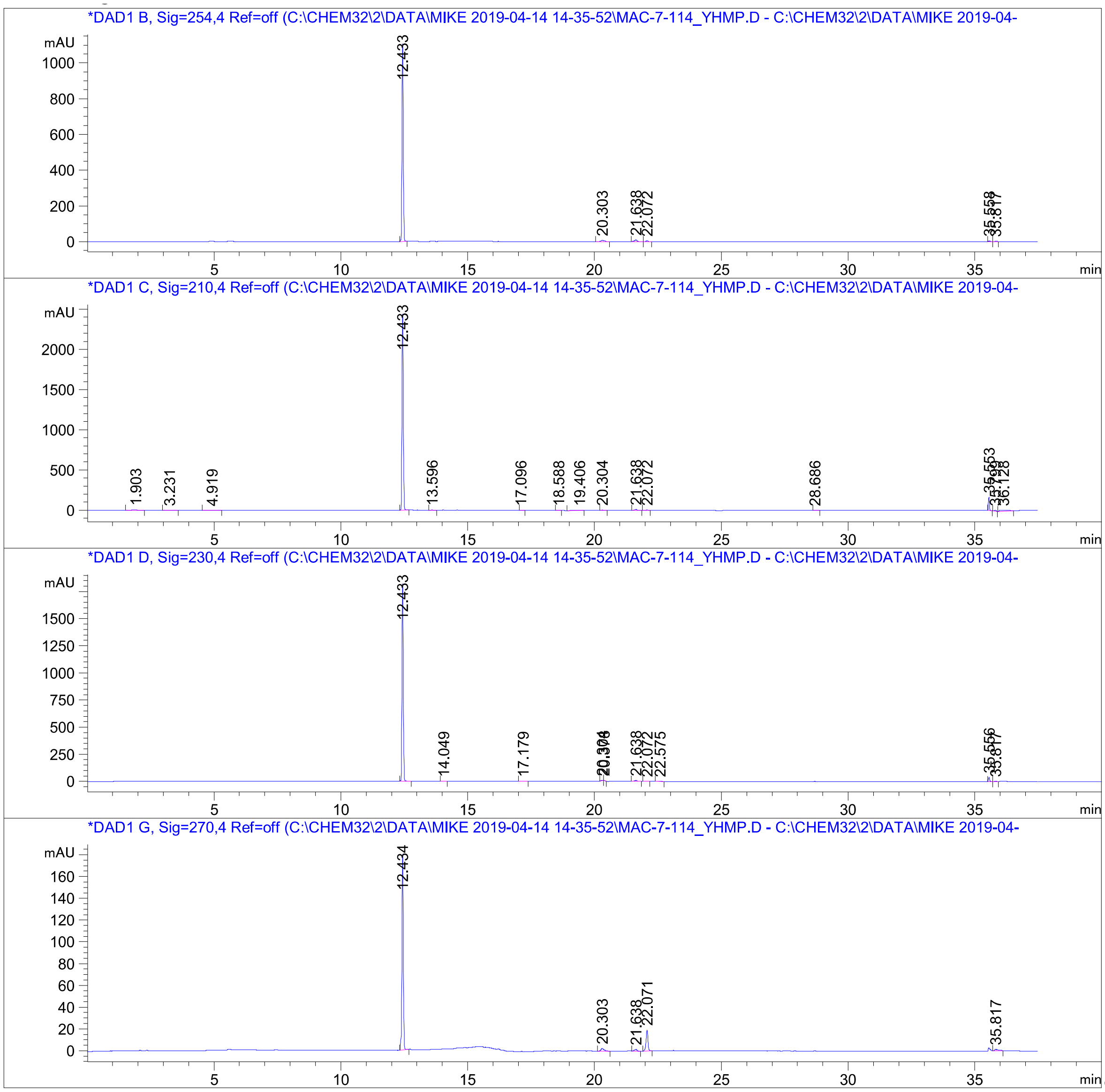

S

upplementary Figure 10. Analytical HPLC trace of Y(III)-HMP-DO3A at 254nm, 210nm, 230nm, and 270nm.

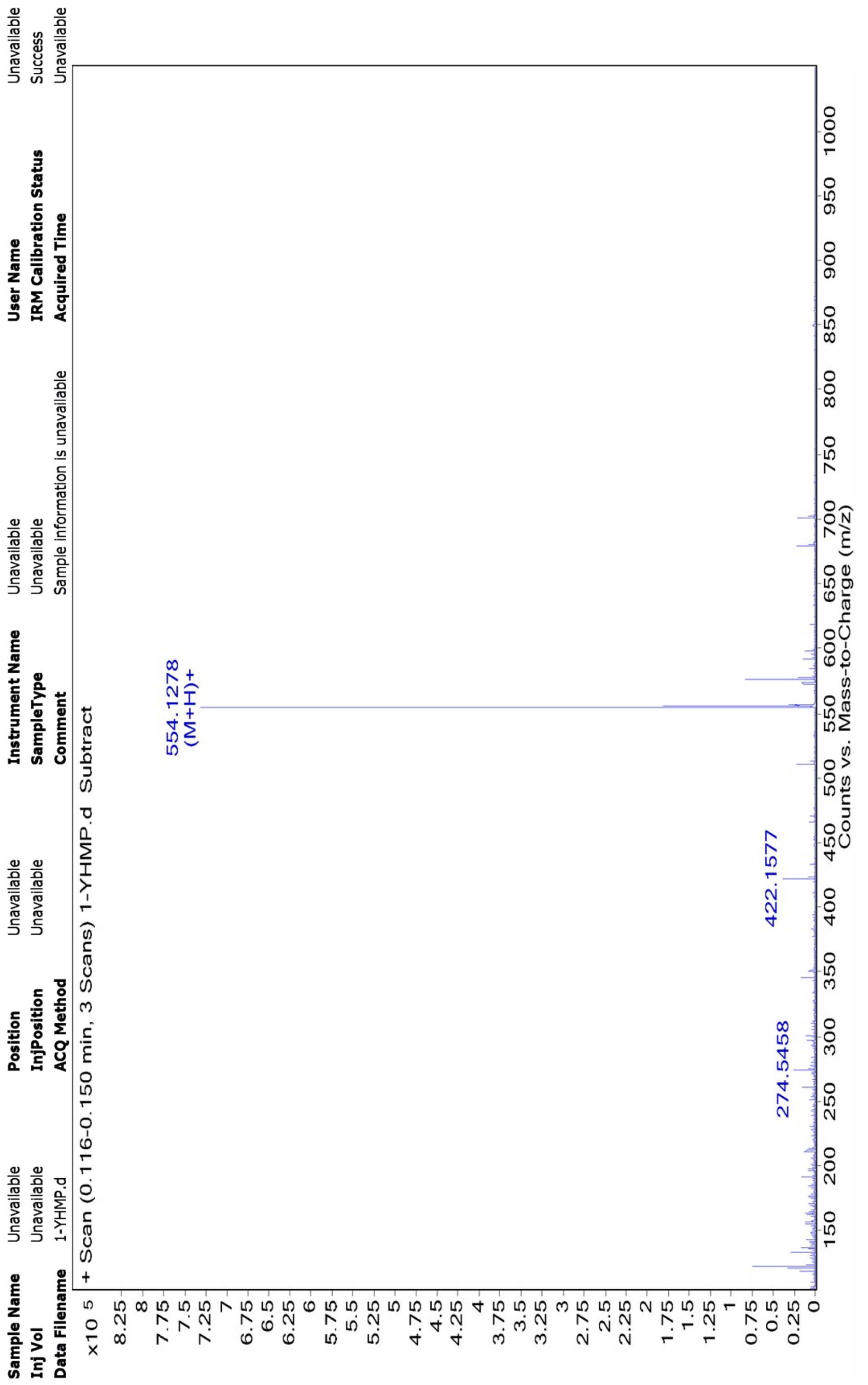

Supplementary Figure 11. HRMS of Y(III)-HMP-DO3A.

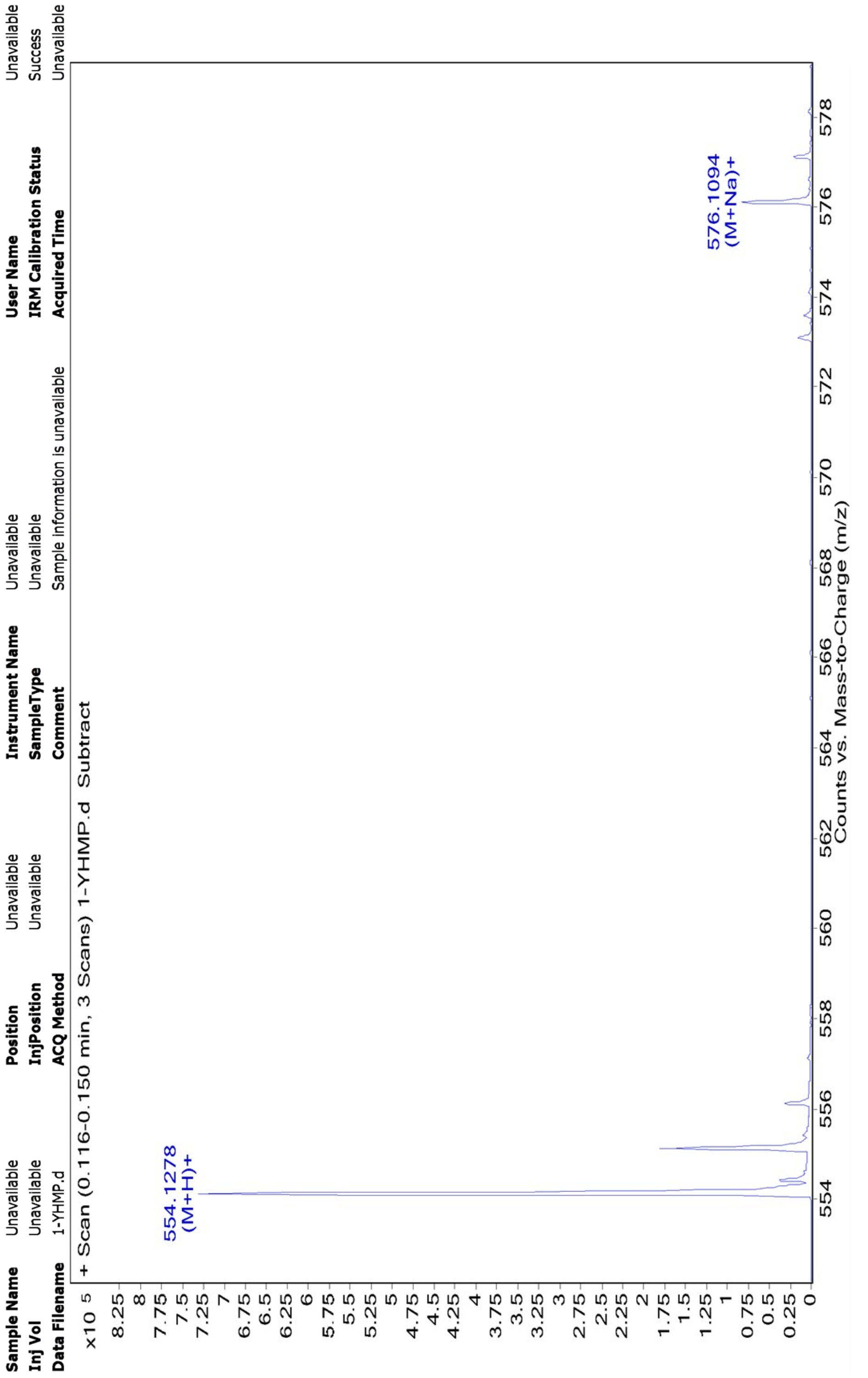

Supplementary Figure 12. HRMS of Y(III)-HMP-DO3A.

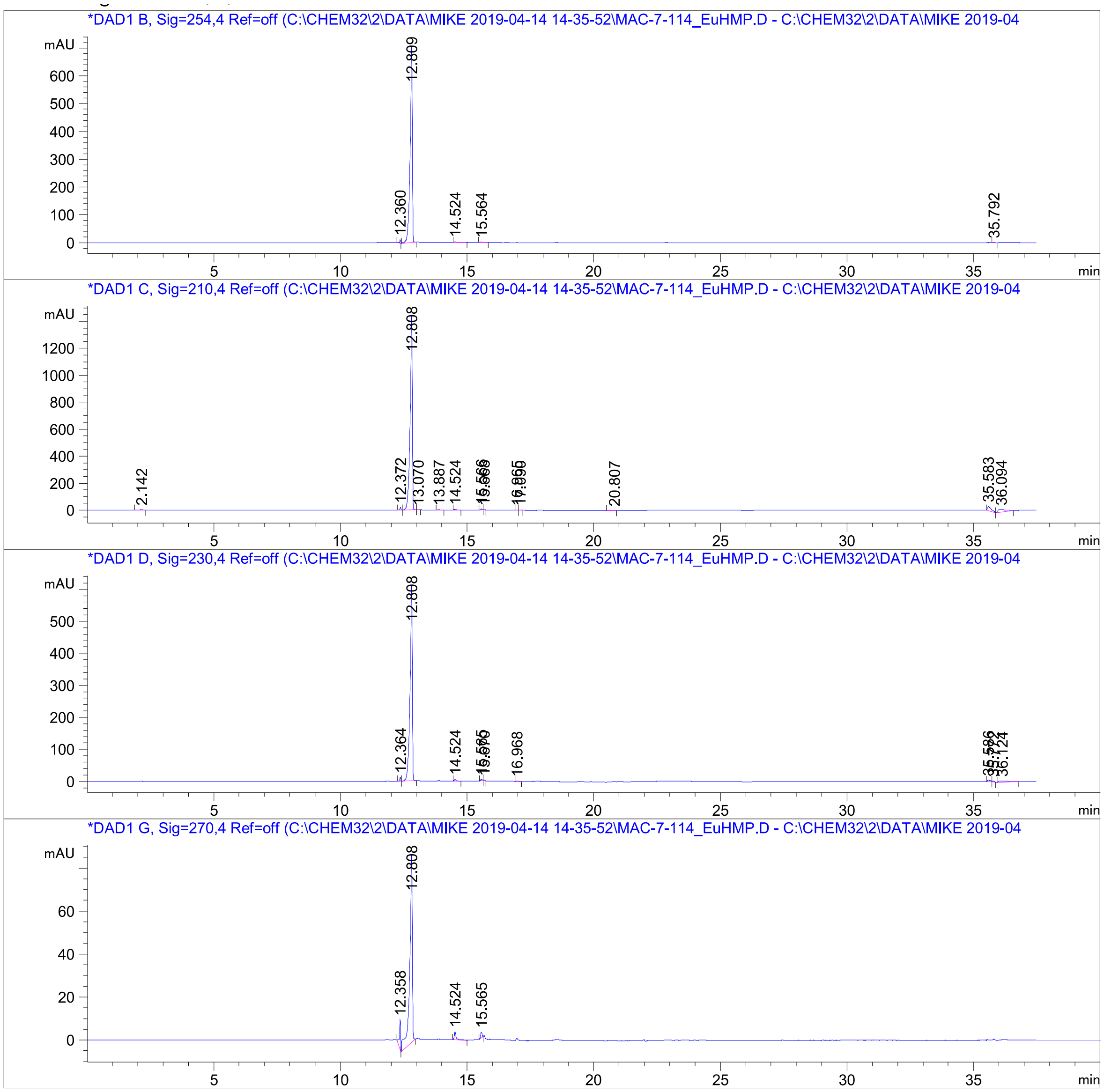

Supplementary Figure 13. Analytical HPLC trace of Eu(III)-HMP-DO3A at 254nm, 210nm, 230nm, and 270nm.

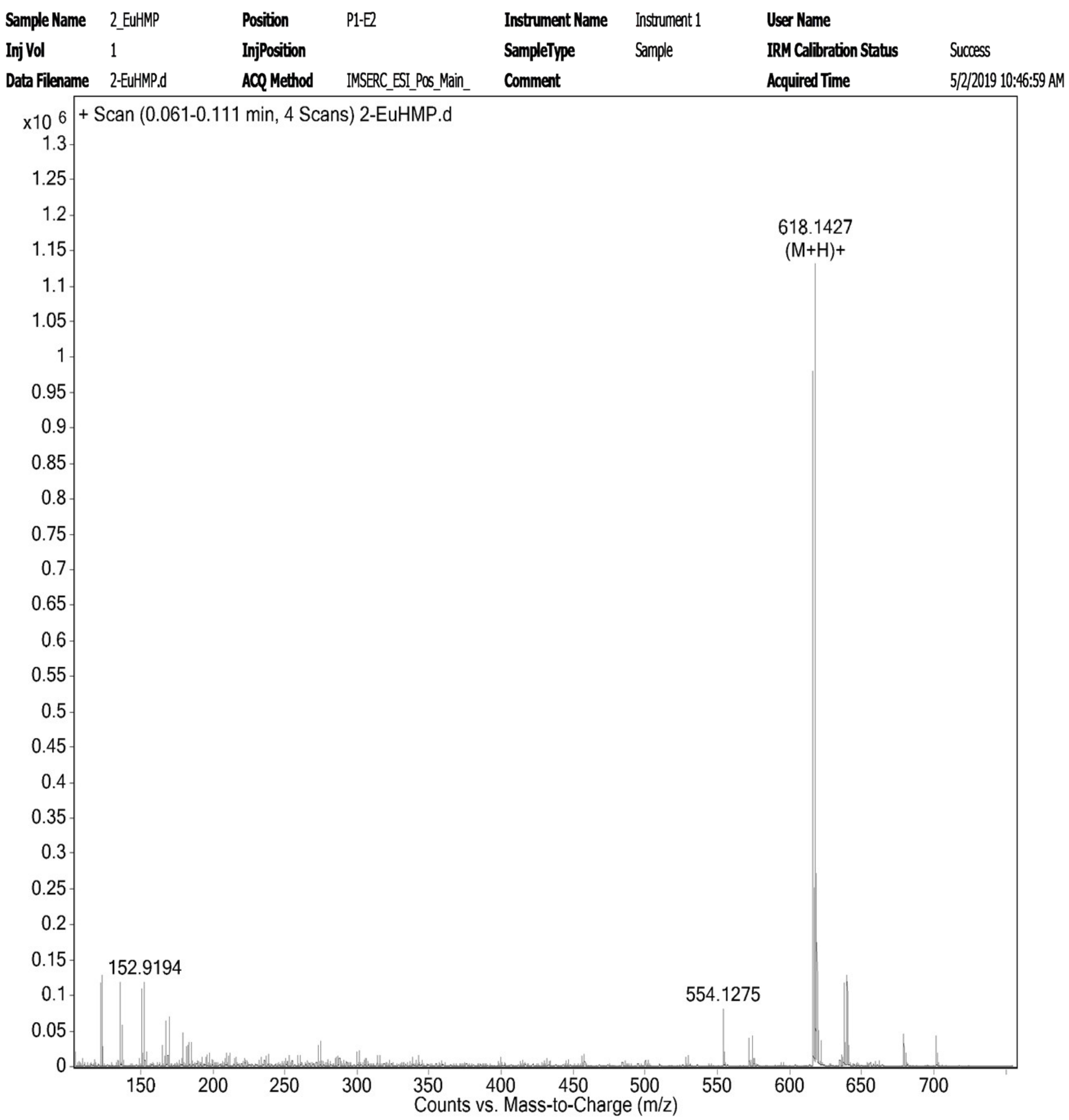

Supplementary Figure 14. HRMS of Eu(III)-HMP-DO3A.

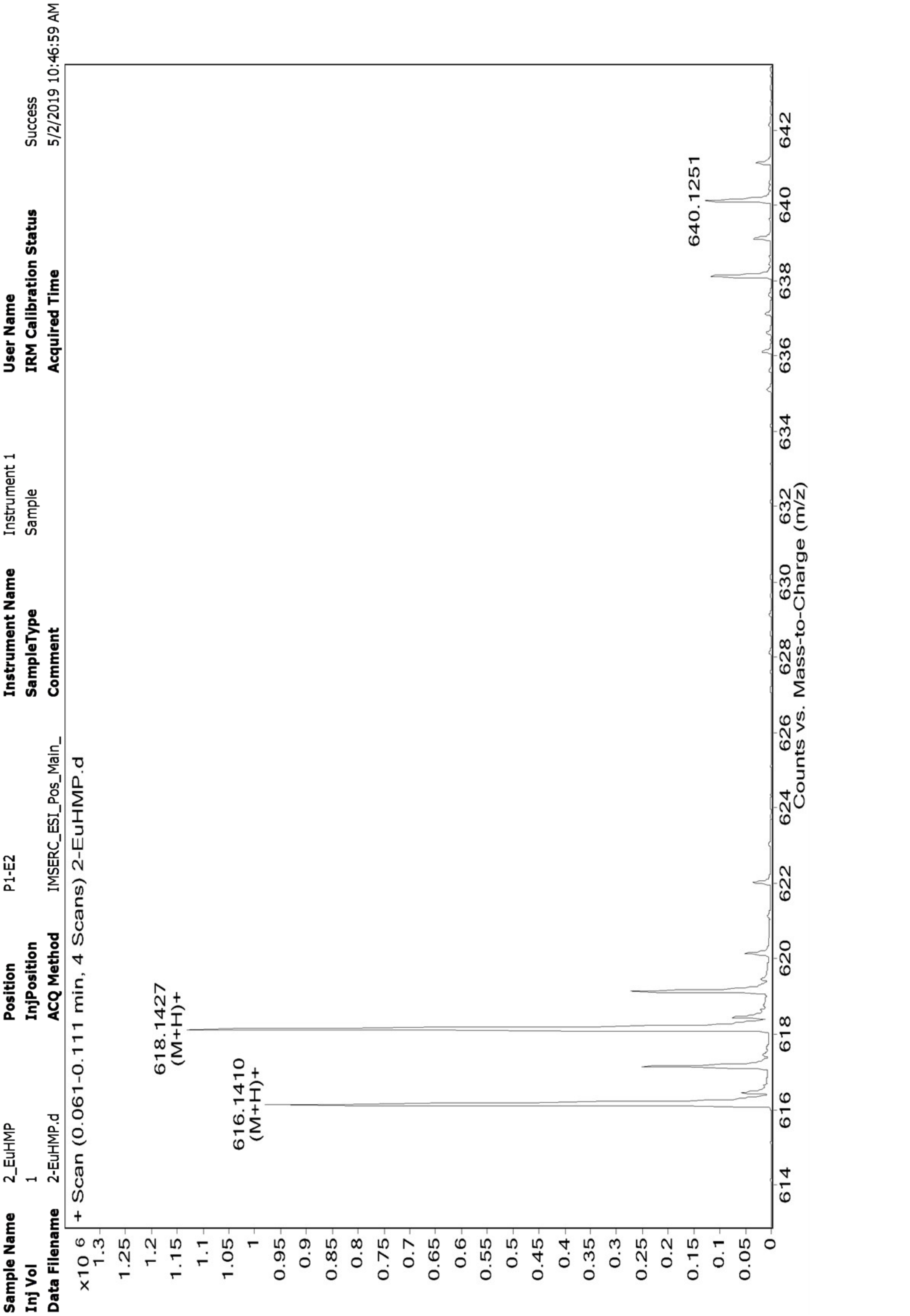

Supplementary Figure 15. HRMS of Eu(III)-HMP-DO3A.
